# Supplementary material for: Ten-Year Trends in Psychotropic Prescribing and Polypharmacy in Australian General Practice Patients with and without Dementia
Source: J Clin Med. 2023 May 10;12(10):3389. doi: 10.3390/jcm12103389 (PMC10219058; doi:10.3390/jcm12103389)

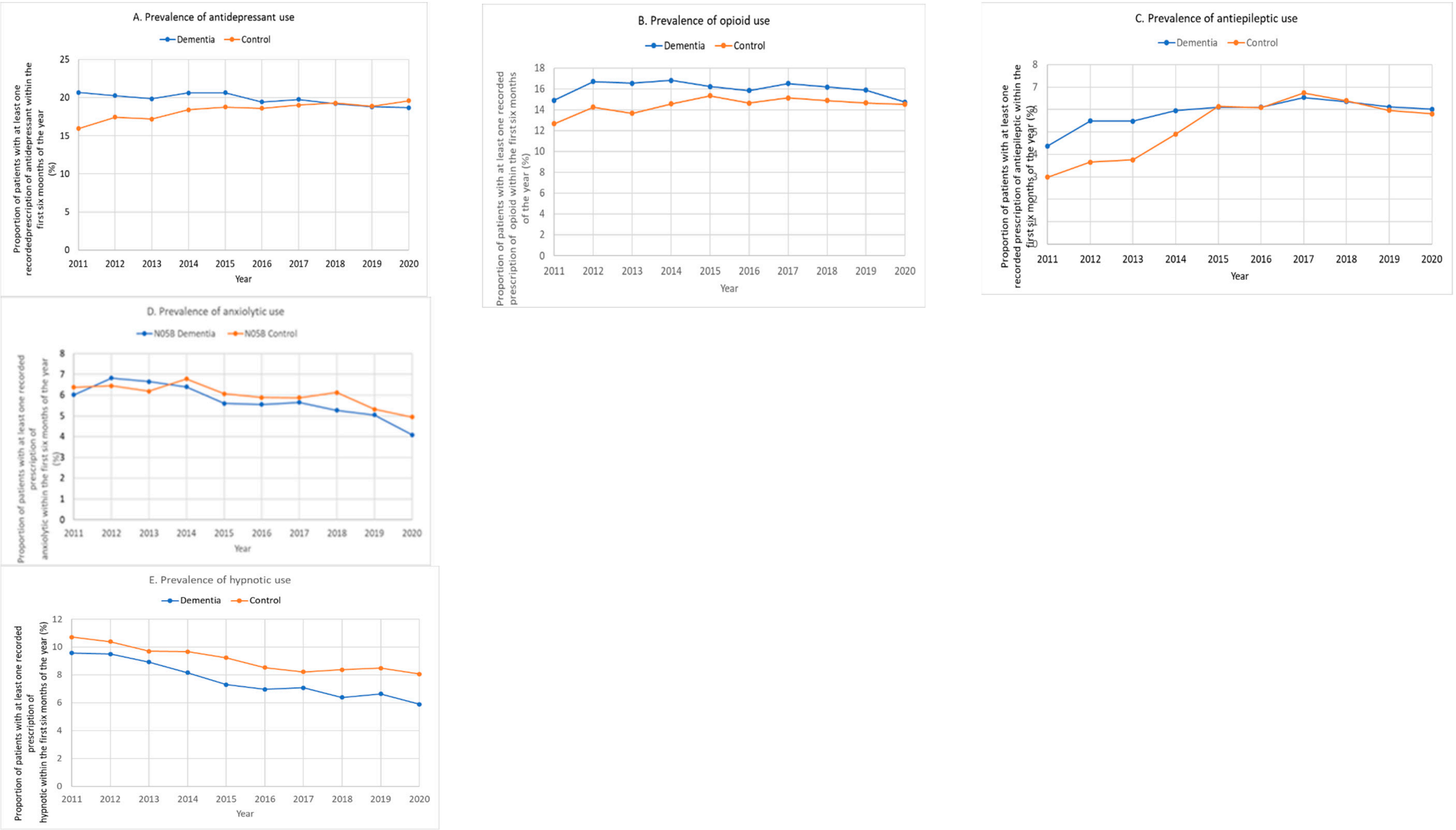

Supplementary Figure S1. A: Trends in prescribing psychotropics in dementia groups and their matched controls: (A) antidepressant use, (B) opioid use, (C) antiepileptic use, (D) anxiolytic use, and (E) hypnotic use.

Supplementary Figure S2. Trends in psychotropic polypharmacy in dementia and control groups without matching

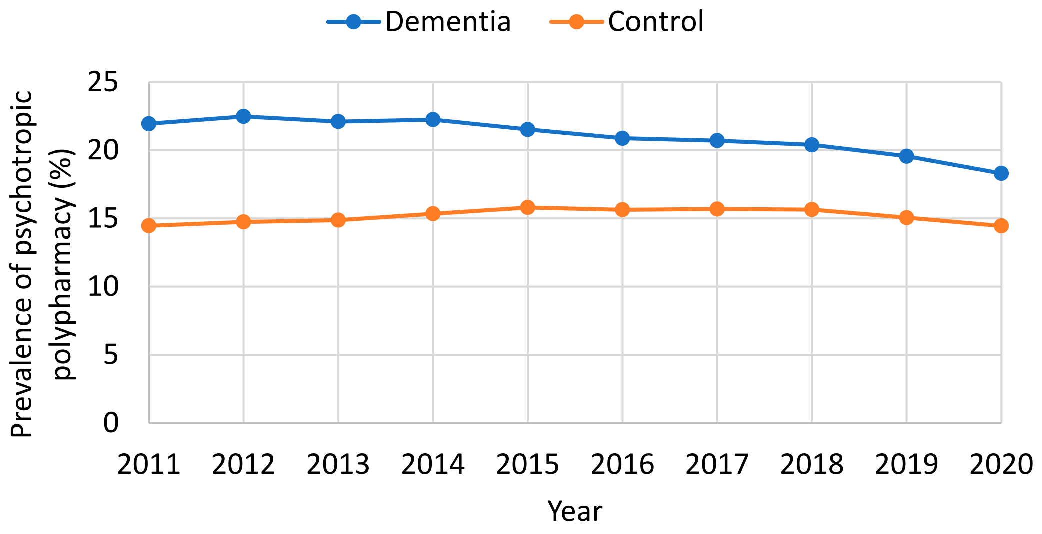

Supplementary Figure S3. Trends in prescribing of psychotropic in the dementia groups aged 85 years or older based on medication class: antidepressant, antipsychotic, opioid, antiepileptic, hypnotic, and anxiolytic

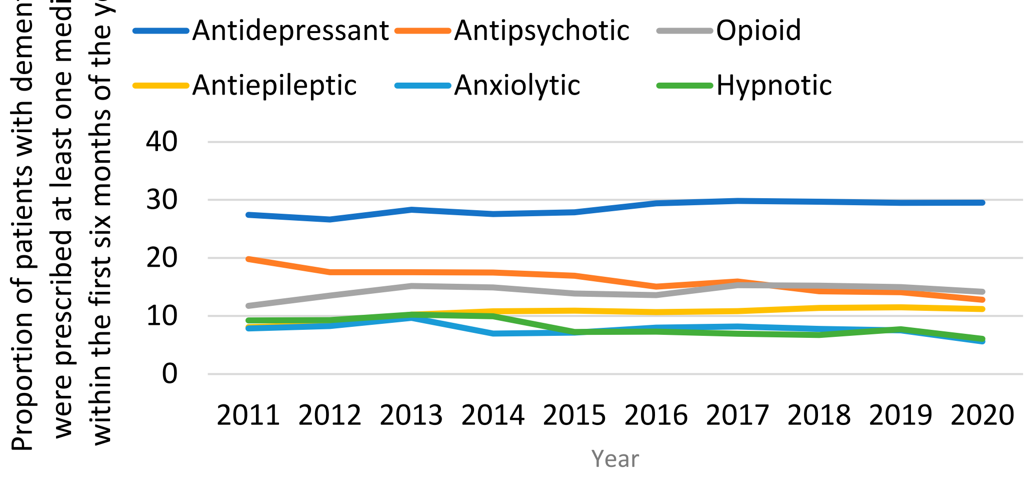

Supplement: Supplementary file 1 [file jcm-12-03389-s001.zip › jcm-2352964-supplementary.pdf]
